# How do you handle post-radiotherapy follow-up care? Results of a national pattern-of-care study

**DOI:** 10.1007/s00066-026-02523-2

**Published:** 2026-04-14

**Authors:** Frank Bruns, Lilli Geworski, Robert M. Blach, Ali Abaci, Hans Christiansen, Jan-Niklas Becker

**Affiliations:** 1 Department of Radiation Oncology, Hannover Medical School (MHH), Carl-Neuberg-Straße 1, 30625 Hannover, Germany; 2 Department of Radiation Protection and Medical Physics, Hannover Medical School, Hannover, Germany

**Keywords:** Post-radiotherapy, Follow-up care, Radiotherapy institutions, Pattern-of-care study, Radiation protection

## Abstract

**Background:**

Post-radiotherapy follow-up care (PFC) is an essential part of radiotherapy and a legal requirement under the German Radiation Protection Act. The aim of this national study was to analyze current PFC structures and concepts in Germany.

**Methods:**

A pattern-of-care study was conducted among all 366 Deutsche Gesellschaft für Radioonkologie (DEGRO)-registered radiotherapy institutions, whereby a 26-item questionnaire on organizational structures, patient selection, follow-up intervals, and the scope of examinations was completed anonymously online.

**Results:**

A total of 95 institutions participated (response rate 26.6%; adjusted 30.2%). Almost all (99%) provide PFC, and 78% offer it regularly for oncology patients. In 84% of the studied institutions, PFC schedules are individually tailored according to the risk of radiation side effects and disease progression. The first follow-up visit occurs within 3 months in 94% of these institutions, and 72% offer PFC for 5 years. In 87% of cases, intervals are shortened for toxicities ≥ grade 3. Frail, very elderly, and palliative patients often receive a reduced PFC program. Telephone-based PFC is performed by 42% and written/digital approaches by 22% of institutions.

**Conclusion:**

Post-radiotherapy follow-up care is firmly established in German radiotherapy practice and is partly tailored to the patient’s individual risk based on radiation side effects and treatment intent. Most institutions adhere to the recommendations of DEGRO and SSK. Digital and structured tools are currently only used to a limited extent in practice. However, they offer considerable potential for future use in further personalizing and optimizing the quality of PFC, also with regard to the still necessary definition and/or harmonization of PFC content.

## Introduction

Post-radiotherapy follow-up care (PFC) is an essential part of radiotherapy and cannot be separated from it conceptually. Post-radiotherapy follow-up care is also a fundamental aspect of the justifying indication (*rechtfertigende Indikation*) for radiotherapy, as it ensures the quality of the results [1]. As the effects of radiation only become apparent in the future and side effects occur randomly over time, it is also medically sensible and necessary to monitor patients who have undergone radiation treatment regularly. This requirement is covered generally by oncology guidelines, but it is described in concrete and detailed terms in radiation protection regulations. In Germany, radiation protection law, e.g., the Radiation Protection Act § 86 (1) No. 15 (StrlSchG) and the Radiation Protection Ordinance § 124 (4) (StrlSchV), obligates radiation oncologists to confirm the long-term success of radiation treatment and thus to carry out PFC [2, 3].

In the 1990s, the German Commission on Radiological Protection (*Strahlenschutzkommission* or *SSK*) established the requirements and recommendations for PFC and subsequently developed these from rigid schemes into an individualized approach. The treating physician should, as far as possible, ensure that the effects and side effects of radiotherapy are recorded and documented using appropriate PFC procedures. Some of the follow-up examinations can be delegated to an appropriately qualified physician, who then forwards the results to the radiation oncologist [4–6].

The present work analyzes the current state of PFC at radiotherapy institutions in Germany to optimize the support of irradiated patients after radiotherapy.

## Materials and methods

A pattern-of-care study consisting of 26 questions, including single-choice, multiple-choice, and free-response questions, was conducted. The topics ranged from basic information such as the organizational structure of the institution to more specific areas such as patient selection, examination intervals, and the extent of the examination.

The commercially available online survey tool “www.umfrageonline.com” was used in this study. The corresponding survey link was sent by email to all Deutsche Gesellschaft für Radioonkologie (DEGRO)-associated institutions across Germany (*n* = 366). In the cover letter, the head of department was asked to either complete the survey or to pass it on to an employee involved in the topic. The survey was available from April 3 to May 25, 2025. The first email was sent on April 3, and a reminder email was sent on April 29, 2025.

Participation in the survey was voluntary and anonymous, and all participants consented to publication of the results. Ethical approval was not required for a survey that utilized an anonymous online questionnaire, and no patient data were collected.

The data were analyzed as follows: the raw data were obtained directly from the online survey tool “www.umfrageonline.com” and further analyzed in Excel (Excel version 2016; Microsoft, Redmond, WA, USA). For analysis, the results were divided into the following three groups: I = university hospital with and without an outpatient health center; II = non-university hospital with and without an outpatient health center; III = outpatient practice with and without an outpatient health center.

Absolute and relative frequencies were calculated for descriptive analyses. This survey was designed as an exploratory, descriptive assessment without predefined hypotheses or formal sample size calculations. Therefore, no statistical hypothesis tests were performed, as group comparisons in an observational study would be prone to multiple testing, limited power, and potentially misleading significance.

## Results

### General information

The DEGRO office provided 366 email addresses of radiation oncology institutions across Germany. However, 9 of these email addresses were found to be non-functional, resulting in a total of 357 valid contacts. Furthermore, there was a duplication of the institution head in 42 email addresses, so it can be assumed that the same procedures apply to PFC in the affiliated institutions. The adjusted collective thus comprises a total of 315 institutions.

**Fig. 1:**
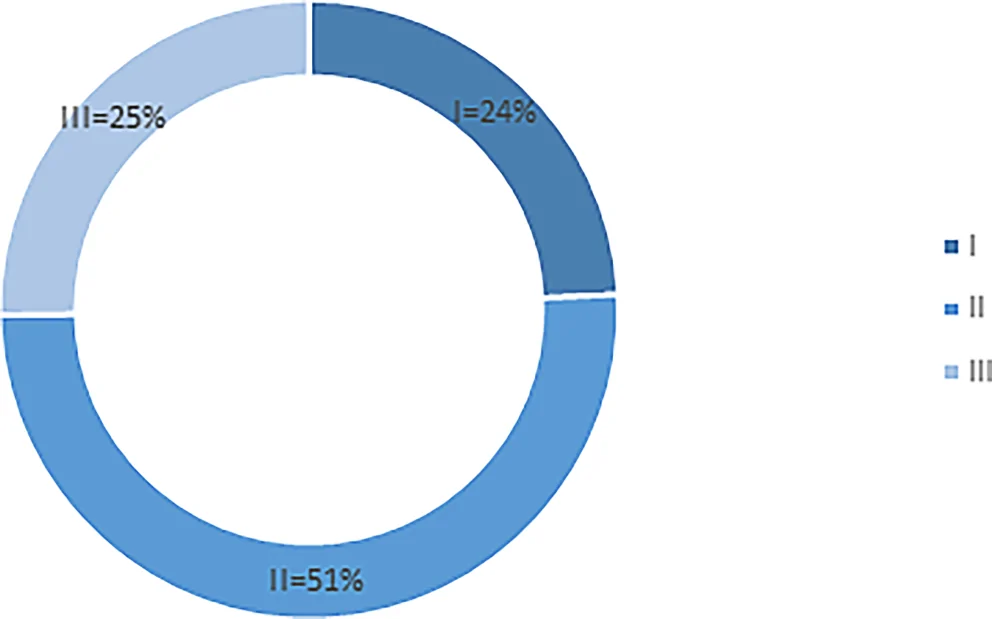
Distribution of the participating institutions (*n* = 95). (I University hospital with/without outpatient health center; II non-university hospital with/without outpatient health center; III outpatient practice with/without outpatient health center)

A total of 95 institutions participated in the survey, resulting in a response rate of 26.6% (95/357) or an adjusted rate of 30.2% (95/315). The characteristics of the participating institutions are listed in Table 1 and shown in Fig. 1. Among the participating institutions, 23 (24%) were type I (university hospitals), 48 (51%) were type II (non-university hospitals), and 24 (25%) were type III (outpatient practice).

**Table 1:** Characteristics of the participating institutions (*n* = 95).

Institution type (multiple choices possible)	Number
I University hospital with/without outpatient health center (MVZ)	23 (24%)
II Non-university hospital with/without outpatient health center (MVZ)	48 (51%)
III Outpatient practice with/without outpatient health center (MVZ)	24 (25%)

### Follow-up offer

Table 2 shows the range of PFC offered, which is further evaluated in subgroups. Almost all studied institutions (*n* = 94/95, 99%) offer PFC for their irradiated patients. Patients undergoing oncological radiotherapy receive regular follow-up care in 70 of the 90 answering institutions (78%). However, some institutions individualize PFC depending on the treatment goal, especially in patients with metastatic disease, taking into account the specific nature of oligometastases in PFC (*n* = 28 for curative treatment vs. *n* = 22 in oligometastatic cases vs. *n* = 12 in palliative treatment for patients with metastatic disease). For some institutions, Eastern Cooperative Oncology Group (ECOG) performance status [7] is an important indicator for limiting PFC. Particularly, an ECOG status of 3 or higher is a limiting factor for 56 of the 89 answering institutions (63%), especially in type II institutions (52.2%) compared to type I (40%) and type III (34.8%) institutions. On the other hand, 27 of the 89 answering institutions (30%) perform PFC regardless of the patients’ ECOG status. Another indicator for limiting PFC is very old age. Twenty of the 86 answering institutions (23%) adjust PFC individually according to the health status and treatment of elderly patients; in some institutions, the age limit is set at 85 years. On the other hand, 66 of the 86 answering institutions (77%) perform PFC regardless of patient age. A further indicator for limiting PFC is if the patient is under legal guardianship. For these patients, PFC is offered on an individual basis by 34 of the 88 answering institutions (39%) and on a regular basis by 52 (59%), while 2 (2%) do not offer them PFC at all. Another indicator for limiting PFC is a language barrier with the patient. For these patients, PFC is offered on an individual basis by 30 of the 89 answering institutions (34%) and on a regular basis by 59 (66%). None of the institutions reject these patients for PFC. Finally, non-malignant disease is a limiting factor for PFC. For patients with non-malignant diseases, 60 of the 91 answering institutions (66%) perform PFC on a regular basis, 24 (26%) perform it on an individual basis, and 31 (34%) do not offer PFC at all. Here, type III institutions most frequently carry out regular follow-up examinations (58.3%), while type II institutions most frequently stated that they did not carry out follow-up examinations for benign diseases (41.3%).

**Table 2:** Post-radiotherapy follow-up care (PFC) offered.

Follow-up offer	All	I	II	III
*Is there a follow up offer?*	95	23	48	33
No	1	–	1 (2.1%)	0
Yes	94	23 (100%)	47 (97.9%)	24 (100%)
*Who is your follow-up care program aimed at? (n* *=* *90)—multiple answers possible*				
All oncology patients	70	15	36	19
Patients receiving curative treatment	28	9	15	4
Oligometastatic patients	22	8	10	4
Metastatic patients	12	4	5	3
Other (e.g., patients with non-malignant disease)	21	5	9	7
*Do you limit follow-up care for frail patients?*	89	20	46	23
No	27	8 (40%)	11 (23.9%)	8 (34.8%)
Yes, from ECOG 2	6	0	3 (6.5%)	3 (13%)
Yes, from ECOG 3	40	8 (40%)	24 (52.2%)	8 (34.8%)
Yes, from ECOG 4	16	4 (20%)	8 (17.4%)	4 (17.4%)
*Do you limit follow-up care for very elderly patients?*	86	20	44	22
No	66	17 (85%)	33 (75%)	16 (72.7%)
Yes	20	–		
– Over 85 years	3	1 (5%)	1 (2.3%)	1 (4.5%)
– Depends on health status and therapy	17	2 (10%)	10 (22.7%)	5 (22.7%)
*How do you handle follow-up care for patients under legal guardianship*	88	20	45	23
Regular follow-up care	52	12 (60%)	27 (60%)	13 (56.5%)
Individual follow-up care services	34	8 (40%)	17 (37.8%)	9 (39.1%)
No follow-up care offered	2	0	1 (2.2%)	1 (4.3%)
*How do you handle follow-up care for patients with language barriers*	89	20	46	23
Regular follow-up care	59	15 (75%)	29 (63.0%)	15 (65.2%)
Individual follow-up care services	30	5 (25%)	17 (37%)	8 (34.8%)
No follow-up care offered	–	0	0	0
*How do you handle follow-up care for patients with benign disease?*	91	21	46	24
Regular follow-up care	36	10 (47.6%)	12 (26.1%)	14 (58.3%)
Individual follow-up care services	24	7 (33.3%)	15 (32.6%)	2 (8.3%)
No follow-up care offered	31	5 (25%)	19 (41.3%)	8 (33.3%)

### Follow-up schedule

**Fig. 2:**
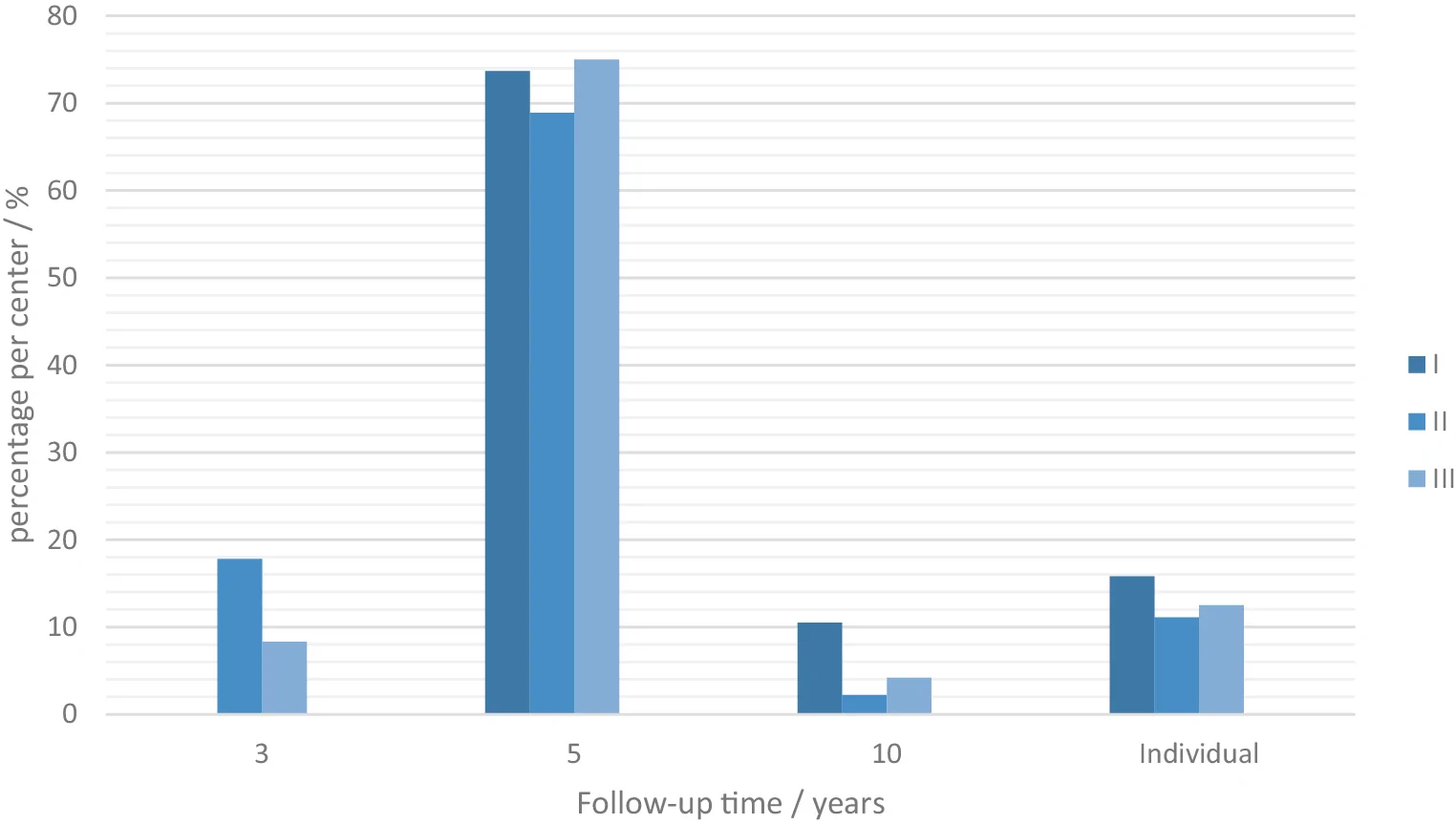
Distribution of follow-up periods (*n* = 88). (I University hospital with/without outpatient health center; II non-university hospital with/without outpatient health center; III outpatient practice with/without outpatient health center)

**Fig. 3:**
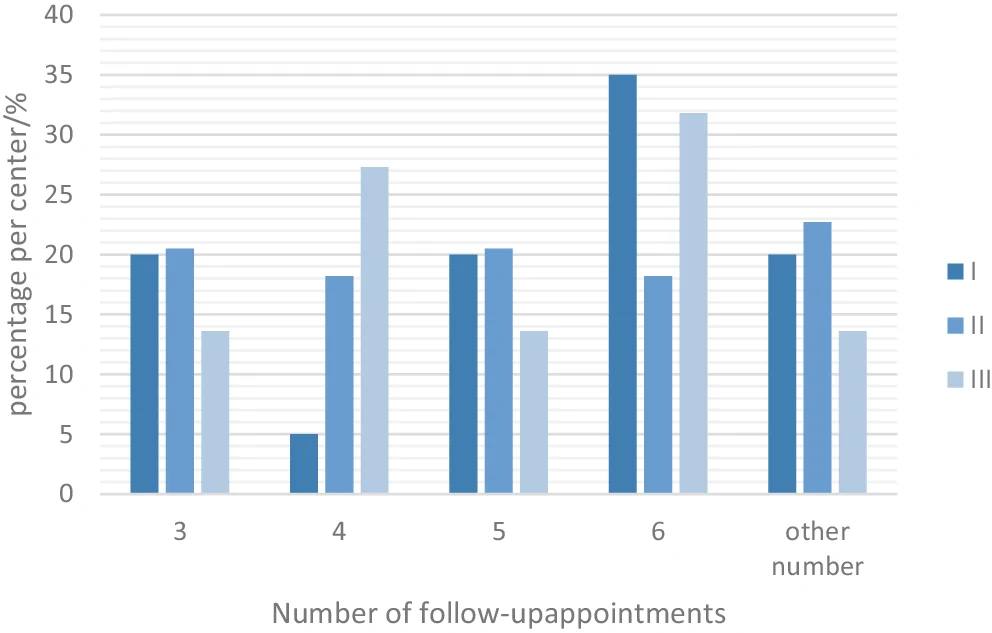
Distribution of number of follow-up appointments (*n* = 86). (I University hospital with/without outpatient health center; II non-university hospital with/without outpatient health center; III outpatient practice with/without outpatient health center)

Table 3 shows the range of PFC schedules, which is further evaluated by subgroup. In general, 79 of the 88 responding institutions (90%) used standard schedules for PFC (94.7% type I, 86.7% type II, and 91.7% type III). The first appointment usually occurs within 3 months (*n* = 85/90, 94%). Further appointments are then made annually in around half of the answering institutions (*n* = 41/85, 48%) and offered over a period of 5 years in around three quarters of the answering institutions (*n* = 63/88, 72%), as shown in Fig. 2. At least 53 of 86 institutions (62%) offer a total of four or more PFC appointments over the entire follow-up period, including 22 of 86 (26%) that offer a total of six PFC appointments (Fig. 3).

**Table 3:** Post-radiotherapy follow-up care (PFC) schedules.

Follow-up schedule	All	I	II	III
*Are patients scheduled for radiation therapy follow-up care according to a standard schedule/rhythm?*	88	19	45	24
Yes	79	18 (94.7%)	39 (86.7%)	22 (91.7%)
No	9	1 (5.3%)	6 (13.3%)	2 (8.3%)
*When does the first appointment after the end of the RT series usually take place? (n* *=* *90)*				
Within 3 months	85	20 (100%)	42 (91.3%)	23 (95.8%)
Within 6 months	3	0	3 (6.5%)	0
After 6 months at the earliest	2	0	1 (2.2%)	1 (4.2%)
*At what intervals are further appointments usually made?*	85	19	43	23
Every 3 months	4	1 (5.3%)	3 (7%)	0
Every 6 months	8	2 (10.5%)	5 (11.6%)	1 (4.3%)
Annually	41	6 (31.6%)	24 (55.8%)	11 (47.8%)
Every 2 years	2	0	2 (4.7%)	0
Alternative intervals	30	10 (52.6%)	9 (20.9%)	11 (47.8%)
*Over what period of time does the follow-up care service usually take place?*	88	19	45	24
Up to 3 years	10	0	8 (17.8%)	2 (8.3%)
Up to 5 years	63	14 (73.7%)	31 (68.9%)	18 (75%)
Up to 10 years	4	2 (10.5%)	1 (2.2%)	1 (4.2%)
Always individually agreed	11	3 (15.8%)	5 (11.1%)	3 (12.5%)
*Cumulatively, the following number of follow-up appointments are usually made*	86	20	44	22
3	16	4 (20%)	9 (20.5%)	3 (13.6%)
4	15	1 (5%)	8 (18.2%)	6 (27.3%)
5	16	4 (20%)	9 (20.5%)	3 (13.6%)
6	22	7 (35%)	8 (18.2%)	7 (31.8%)
Other number	17	4 (20%)	10 (22.7%)	3 (13.6%)
*Does your department/practice customize the follow-up care plan?*	88	20	44	24
Yes	74	18 (90%)	36 (81.8%)	20 (83.3%)
No	14	2 (10%)	8 (18.2%)	4 (16.7%)
*Does the appointment schedule for your patients differ, e.g., according to the risk of radiation effects?*	88	20	44	24
Yes	73	18 (90%)	38 (86.4%)	17 (70.8%)
No	15	2 (10%)	6 (13.6%)	7 (29.2%)
*Does the appointment schedule for your patients differ, e.g., according to the risk of disease progression?*	88	20	44	24
Yes	54	15 (75%)	28 (63.6%)	11 (45.8%)
No	34	5 (25%)	16 (36.4%)	13 (54.2%)
*Do you shorten the interval between appointments for your patients (on an individual basis) if serious side effects occur (≥* *grade 3)?*	87	20	43	24
Yes	76	17 (85%)	37 (86%)	22 (91.7%)
No	11	3 (15%)	6 (14%)	2 (8.3%)
*Are there patients who are deliberately scheduled for only a single follow-up (n* *=* *88)—multiple answers possible*				
No	16	3	10	3
Palliative patients	41	8	21	12
Metastatic patients	15	3	7	5
Patients with non-malignant disease	35	9	15	11
Frail patients	35	6	19	10
Patients under legal guardianship	7	2	4	1
Patients with language barriers	5	1	3	1
Other	19	5	6	8

**Fig. 4:**
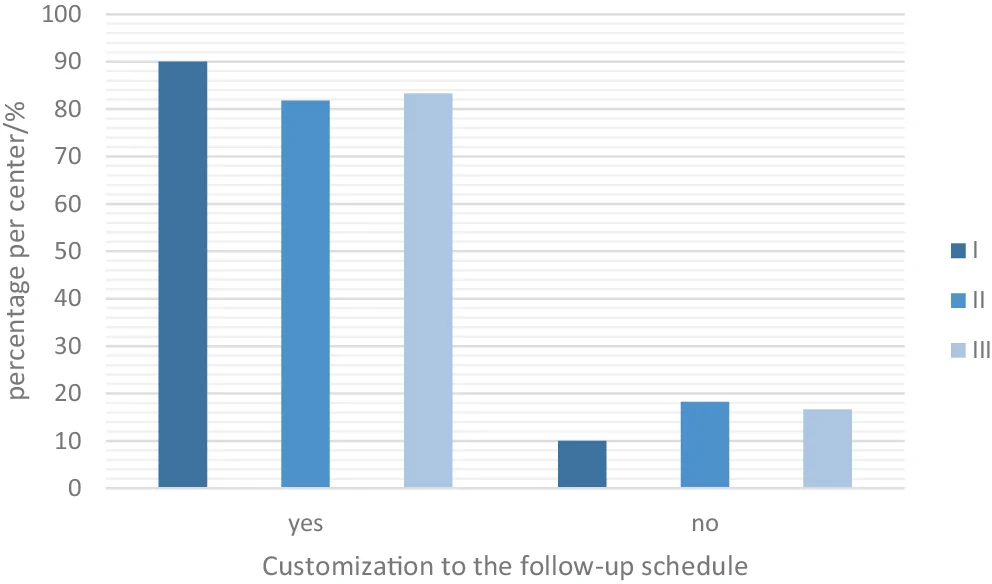
Distribution of customization to the follow-up schedule (*n* = 88). (I University hospital with/without outpatient health center; II non-university hospital with/without outpatient health center; III outpatient practice with/without outpatient health center)

Out of 88 institutions, 74 (84%) customize the PFC schedule, while 14 (16%) do not (Fig. 4). In 73 of 88 institutions (83%), individualization is based on the risk of radiation effects; furthermore, in 54 of 88 institutions (61%), it is also based on the risk of disease progression. In the event of serious radiation side effects (grade 3 or higher), 76 of 87 institutions (87%) offer individual patients shorter intervals between PFC appointments. On the other hand, certain patient groups only receive a one-time offer of PFC: 56 of 88 institutions (64%) cite a (clear) palliative goal of radiotherapy as the reason, 35 of 88 institutions (40%) cite the frailty of the patient, and 35 of 88 institutions (40%) cite radiotherapy for benign diseases, while in another 31 of 88 cases (35%), other reasons are given; 16 of 88 institutions (18%) refuse to offer only a one-time PFC examination.

### Follow-up procedures

Table 4 shows the range of PFC procedures. A total of 83 of the 85 institutions (98%) that participated in the survey stated that PFC examinations are usually performed after a personal appointment has been made, as further detailed in Table 4. If the patient does not attend, 66 of the 87 responding institutions (76%) send a reminder by telephone or in writing (84.2% type I, 72.7% type II, and 75% type III).

**Table 4:** Post-radiotherapy follow-up care (PFC) procedures.

Follow up procedures	All	I	II	III
*How do you provide (regular) follow-up care? (n* *=* *85)—multiple answers possible*				
Personal appointment	83	19	42	22
By telephone	36	10	14	12
In writing via questionnaire (analog)	17	3	7	7
In writing via app (digital)	2	1	0	1
*Are patients who do not attend a follow-up appointment reminded of the appointment by telephone/in writing, or are they asked why they did not attend?*	87	19	44	24
Yes	66	16 (84.2%)	32 (72.7%)	18 (75%)
No	21	3 (15.8%)	12 (27.3%)	6 (25%)
*Does your (regular) follow-up care include a medical history?*	87	19	44	24
Always	73	17 (89.5%)	36 (81.8%)	20 (83.3%)
Most of the time	11	2 (10.5%)	6 (13.6%)	3 (12.5%)
Occasionally	2	0	2(4.5%)	0
Never	1	0	0	1(4.2%)
*Does your (regular) follow-up care include a clinical examination?*	87	19	44	24
Always	35	10 (52.6%)	14 (31.8%)	11 (45.8%)
Most of the time	41	8 (42.2%)	25 (56.8%)	8 (33.3%)
Occasionally	11	1 (5.3%)	5 (11.4%)	5 (20.8%)
Never	0	0	0	0
*Does your (regular) follow-up care include an assessment of follow-up imaging, if available?*	87	19	44	24
Always	58	15 (78.9%)	32 (72.7%)	11 (45.8%)
Most of the time	20	3 (15.8%)	7 (15.9%)	10 (41.7%)
Occasionally	8	1 (5.3%)	4 (9.1%)	3 (12.5%)
Never	1	0	1 (2.3%)	0
*Do you or your department arrange for further diagnostic examinations as part of follow-up care?*	87	19	44	24
No	24	4 (21.1%)	11 (25%)	9 (37.5%)
Yes, ultrasound	1	1 (5.3%)	0	0
Yes, further CT imaging	22	3 (15.8%)	15 (34.1%)	4 (16.7%)
Yes, further MRI imaging	4	3 (15.8%)	0	1 (4.2%)
Other	36	8 (42.1%)	18 (40.9%)	10 (41.7%)
*Are higher-grade toxicities recorded in a separate list/file?*	88	19	45	24
Yes	49	9 (47.4%)	24 (53.3%)	16 (66.7%)
No	39	10 (52.6%)	21 (46.7%)	8 (33.3%)
*Is there a note in the doctor’s letter (final letter) requesting notification and/or follow-up if RT side effects occur?*	87	19	45	23
Yes	76	18 (94.7%)	37 (82.2%)	21 (91.3%)
No	11	1 (5.3%)	8 (17.8%)	2 (8.7%)

The regular PFC examination includes a medical history check in 84 of 87 institutions (97%), a clinical examination in 76 of 87 institutions (87%), and a medical imaging check in 78 of 87 institutions (90%). Imaging assessment was “always” performed in type I and II institutions in 78.9% and 72.7% of cases, respectively. Type III institutions more frequently stated that they only performed the assessment “most of the time.” Further diagnostic examinations are carried out or were arranged by the radiotherapy department as part of the PFC examination regularly in 27 of 87 institutions (31%; as further detailed in Table 4), individually for specific issues in 36 of 87 institutions (41%), and never in 24 of 87 institutions (28%).

Higher toxicities are also recorded in a separate special list in 49 of the 88 answering institutions (56%). Of the 87 answering institutions, 76 (87%) request notification and/or follow-up examination if side effects of radiation therapy occur in the doctor’s final letter.

## Discussion

To our knowledge, this survey is the first of its kind conducted in radiotherapy institutions in Germany. The primary aim was to assess their adherence to modern approaches to post-radiotherapy follow-up care (PFC) and to identify factors associated with the utilization of follow-up care. A total of 95 institutions participated in the survey, amounting to a response rate of 26.6% (95/357) or an adjusted rate of 30.2% (95/315), including 49 hospitals and 78 outpatient practices/outpatient health centers.

Almost all of the institutions surveyed (*n* = 94/95, 99%) offer PFC to their patients. Patients undergoing radiotherapy for malignant disease receive regular follow-up care in 78% of the institutions (*n* = 70/90), indicating a high degree of consensus among radiotherapy experts that patients who have undergone radiotherapy should be offered this form of follow-up care. However, some of this consensus stems from the obligation under radiation protection legislation stating that radiation therapists must offer PFC.

This obligation to carry out PFC was first introduced in 1992, after which the German Commission on Radiological Protection (SSK) specified the requirements for PFC in its recommendations of 1998 and 2002. According to these recommendations, follow-up examinations after radiotherapeutic procedures of malignant tumors should take place at defined times, e.g., at annual intervals over 5 years [4–6]. Including an initial follow-up examination 3 months after radiotherapy, the PFC program comprises a total of six appointments throughout the entire follow-up period. Although the current SSK recommendations are not directly legally binding, they nevertheless serve as clear guidelines due to the authority of the publishing actors (Table 5). For monitoring purposes, so-called competent authorities (*ärztliche Stellen*) were introduced, which, in accordance with the directive on radiation protection in medicine (*Richtlinie Strahlenschutz in der Medizin*), can request documents from every radiotherapy institution in Germany, e.g., reports on acute and chronic radiation side effects and on the results of PFC, to verify the success of treatment [8].

**Table 5:** Legally binding force of regulatory measures.

Regulations	Commitment
Constitution	Generally binding
Atomic Energy Act	
Radiation Protection Act	
Radiation Protection Ordinance	
General administration regulations	Binding for authorities
Guideline	Binding by specification in the license or by supervisory measures
Recommendations of the German Commission on Radiological Protection (SSK)	
DIN standards	
Technical specifications	
In-house regulations	

The recommendations of the SSK were re-evaluated in 2011 based on new scientific findings and amended so that a rigid schedule for PFC could be abandoned in favor of an individual, risk-based approach. Patients could now be classified according to their risk of radiation side effects (high, medium, or low). The three resulting groups are cared for with varying degrees of intensity and personal engagement by specialized radiation oncologists during the PFC process [9–11]. Subsequently, the DEGRO, the responsible professional association in Germany, published follow-up care recommendations in 2015 [12].

In this study, 84% of institutions (*n* = 74) reported customizing their PFC schedules individually. The individualization of PFC is considered particularly important in cases of metastatic disease, frail patients (ECOG status ≥ 3), very elderly patients, patients under legal guardianship, patients with language barriers, and in cases of benign diseases, and is therefore offered accordingly. Individualization is frequently achieved by reducing the number of PFC examinations, as suggested in the DEGRO recommendations. Here, variation of the PFC intervals from the annual appointment (*n* = 44, 52%) is used significantly more frequently than variation from the 5‑year period (*n* = 25, 28%). On the other hand, 26% of institutions (*n* = 22) still offer PFC programs with a total of six appointments during the 5‑year follow-up period, in line with older recommendations from 1998/2002. Reasons for more frequent PFC appointments could include, for example, consistent data collection in the context of health care research [13–16].

Certain patient groups are only reappointed to PFC once. The DEGRO recommendations explicitly permit this as the lowest possible PFC offer after palliative radiotherapy and after treatment of benign diseases. The reasons given in the survey include palliative radiotherapy (64%, *n* = 56 institutions), frailty of the patients (40%, *n* = 35), radiotherapy for benign diseases (40%, *n* = 35), and other reasons (35%, *n* = 31). A total of 18% of institutions (*n* = 16) refuse to offer only a one-time PFC examination. However, from a clinical practice perspective, it is questionable whether palliative care patients in particular should be excluded from regular PFC, as this could delay the detection of clinical progression, which could however necessitate retreatment within a few months. It should also be noted that when low radiation doses are administered for benign conditions, approximately 30% of patients benefit from a second course of radiotherapy, as reported by Hautmann et al. using the example of painful osteoarthritis [17]. Therefore, it is advisable to administer at least one course of PFC in this case. On the other hand, PFC examinations are routinely intensified on an individual basis in patients with serious side effects in 87% of institutions (*n* = 76). Radiation oncologists feel strongly committed to continuing to care for these patients, which aligns with the DEGRO recommendations. Since the timing of the onset of radiation effects is uncertain, the doctor’s final letter from 87% of the institutions (*n* = 76) states that notification and/or follow-up examination by the radiotherapy specialist is required if side effects occur. Higher-graded toxicities are additionally recorded in a separate list/file by 56% of the institutions (*n* = 49). These lists/files are usually compiled on the recommendation of competent authorities.

To date, there is little literature on the individualization of PFC that practicing radiation oncologists can use as a resource. Therefore, the DEGRO published follow-up care recommendations in 2015 [12]. Scientific studies on the possible consequences of individualization of PFC remain scarce.

To investigate the effects of reducing the number of PFC appointments in patients with breast cancer, Holtgrave et al. [18] conducted a retrospective longitudinal observational study. Postoperative radiotherapy for breast cancer is considered a classic example of radiotherapy with a low risk of radiation side effects. Two selected cohorts of breast cancer patients (*n* = 192) with different PFC plans over 5 years were retrospectively compared. No differences were found in terms of follow-up events and the long-term effects of radiation therapy, indicating that reducing follow-up examinations to four over 5 years for breast cancer patients is similarly effective to conventional follow-up care with six appointments over 5 years.

A total of 98% of institutions (*n* = 83) stated that PFC examinations are usually performed after a personal appointment has been made. The regular PFC examination includes a medical history check in 97% of cases, a clinical examination in 87% of cases, and a medical imaging check in 90% of cases in the institutions surveyed. If the patient does not attend, 76% of institutions (*n* = 66) send a reminder by telephone or in writing. This is an important and effective countermeasure that can increase patient adherence from around 80% to up to about 95%, as Lange et al. [19] recently demonstrated in PFC patients with breast or prostate cancer.

A total of 42% of institutions (*n* = 36) state that they conduct PFC by telephone, while a further 22% (*n* = 19) use questionnaires or apps. This indicates a comparatively low degree of individualization. Although the DEGRO recommendations include telephone interviews as one possible option for follow-up documentation, they do not identify this modality as the preferred approach for patients with a low to moderate risk after radiotherapy with a sufficiently established and generally accepted treatment protocol. However, one reason for this could be that apps such as electronic patient-reported outcome systems (ePRO), which could be used for PFC in radiation oncology, are unfortunately rarely available to patients and healthcare professionals on common marketplaces and are not yet mature enough for routine clinical practice [20].

Our survey focuses on the framework conditions of PFC. However, optimizing PFC also requires a discussion of its content. Institutions such as DEGRO, with its various working groups, could be a good platform to provide standardized protocols and questionnaires for optimizing the follow-up care of patients in radiation oncology in the future. In addition, systematic data collection within the framework of the PFC, combined with the (systematic) use of epidemiological cancer registry data, would open up new perspectives for clinical medicine and support the further development and clinical evaluation of innovative therapeutic approaches [21].

This survey, which focuses on the structures for PFC in radiotherapy institutions in Germany, is restricted by certain limitations. One of the greatest limitations of a pattern of care study is the low response rate of completed data, which also correlates with the size of the collective surveyed. Our survey achieved a good response rate of around 30%, which remains sufficiently representative of the relevant population. Nevertheless, the results of this predominantly descriptive study should be interpreted with caution [22]. By including all types of healthcare structures/institutions, the entire spectrum of PFC approaches can be represented. The anonymity of the survey generally enables more honest answers to the questions, which is another strength of this survey. In addition, interest in the topic and an appropriate number of questions (*n* = 26) likely contributed to its success. Further limitations were that due to the anonymity of the survey, it was not possible to narrow down the figures further. The number of affiliated institutions has also increased significantly over the last 10 years; thus, the comparative figure for institutions is likely to be well below 324, which should in turn increase the significance of our results.

## Conclusion

This survey provides the first nationwide overview of the current structures and practices of post-radiotherapy follow-up care (PFC) in Germany. In summary, this survey shows a broad consensus among radiation oncology institutions in Germany that PFC should be provided. Most participating institutions reported adapting their PFC schedules, although the specific criteria and extent of customization varied. According to the responses, adaptations were implemented across different patient groups, including those with reduced general health status, metastatic disease, or benign conditions. One-time follow-up examinations were used in selected situations, as reflected in the questionnaire, although clear criteria for such decisions were not uniformly reported. In palliative patients, however, it should be remembered that exclusion from regular PFC may delay the detection of clinical progression, which could however necessitate retreatment within a few months. In our survey, a minority of institutions indicated that they do not offer PFC for non-malignant diseases, highlighting heterogeneous practice patterns in this area. Personal appointments remain the predominant format for PFC, while telephone- or questionnaire-based approaches are used less frequently. These findings indicate that although alternative formats are available in some centers, further research is needed to determine how different elements of PFC can be safely and effectively individualized across different patient groups in a consistent and evidence-based manner. This survey addresses the framework of PFC, but its optimization also requires definition of its content. Organizations such as DEGRO could serve as platforms for developing standardized protocols and questionnaires to optimize follow-up care among radiation oncology patients in the future.

## Data Availability

The datasets used and/or analyzed during the current study are available from the corresponding author upon reasonable request.
